# Effects of contact structure on the transient evolution of HIV virulence

**DOI:** 10.1371/journal.pcbi.1005453

**Published:** 2017-03-31

**Authors:** Sang Woo Park, Benjamin M. Bolker

**Affiliations:** 1 Department of Mathematics & Statistics, McMaster University, Hamilton, Ontario, Canada; 2 Department of Biology, McMaster University, Hamilton, Ontario, Canada; 3 Institute for Infectious Disease Research, McMaster University, Hamilton, Ontario, Canada; ETH Zurich, SWITZERLAND

## Abstract

Early in an epidemic, high densities of susceptible hosts select for relatively high parasite virulence; later in the epidemic, lower susceptible densities select for lower virulence. Thus over the course of a typical epidemic the average virulence of parasite strains increases initially, peaks partway through the epidemic, then declines again. However, precise quantitative outcomes, such as the peak virulence reached and its timing, may depend sensitively on epidemiological details. Fraser *et al.* proposed a model for the eco-evolutionary dynamics of HIV that incorporates the tradeoffs between transmission and virulence (mediated by set-point viral load, SPVL) and their heritability between hosts. Their model used implicit equations to capture the effects of partnership dynamics that are at the core of epidemics of sexually transmitted diseases. Our models combine HIV virulence tradeoffs with a range of contact models, explicitly modeling partnership formation and dissolution and allowing for individuals to transmit disease outside of partnerships. We assess summary statistics such as the peak virulence (corresponding to the maximum value of population mean log_10_ SPVL achieved throughout the epidemic) across models for a range of parameters applicable to the HIV epidemic in sub-Saharan Africa. Although virulence trajectories are broadly similar across models, the timing and magnitude of the virulence peak vary considerably. Previously developed implicit models predicted lower virulence and slower progression at the peak (a maximum of 3.5 log_10_ SPVL) compared both to more realistic models and to simple random-mixing models with no partnership structure at all (both with a maximum of ≈ 4.7 log_10_ SPVL). In this range of models, the simplest random-mixing structure best approximates the most realistic model; this surprising outcome occurs because the dominance of extra-pair contact in the realistic model swamps the effects of partnership structure.

## Author summary

Pathogens such as HIV can evolve rapidly when the environment changes. One important aspect of a pathogen’s environment is the probability that an infectious contact (a sneeze for a respiratory disease, or an unprotected sex act for a sexually transmitted disease) encounters an uninfected person and thus has a chance to transmit the pathogen. As an epidemic grows the number of uninfected people shrinks, changing evolutionary pressures on the pathogen. While researchers have used models to explore pathogen evolution during epidemics, their models usually neglect important processes such as people entering and leaving sexual partnerships. We compared several evolutionary models for HIV that include partnership dynamics as well as sexual contact outside of stable partnerships. Models of intermediate complexity predicted lower virulence midway through the epidemic (a minimum of 15 years to progress to AIDS) than either more realistic models or simple models with no partnership structure (both with a minimum of 7.25 years to progress to AIDS), because random sexual contacts tended to wash out the effects of stable partnerships. Researchers trying to predict the evolution of pathogens must try to understand the implications of their modeling choices; models of intermediate complexity may not produce intermediate conclusions.

## Introduction

The evolution of pathogen virulence (the harm done to the pathogen’s host) has both theoretical and, potentially, practical importance. Evolutionary theory suggests that pathogens with higher reproduction numbers ($R_{0}$)—the number of secondary infections caused by a single infected host over the course of its infectious period—will increase in prevalence relative to strains with lower reproductive ratios. Pathogens can increase their reproduction numbers either by increasing their transmission rate, the rate (per infected host) at which they infect new hosts, or by decreasing their clearance or disease-induced mortality rate, the rate at which hosts recover or die from disease. The *trade-off theory* [1] postulates that transmission and disease-induced mortality rates are both driven by the rate at which the pathogen exploits host resources for within-host reproduction, and that pathogen evolution will thus strike a balance between the pathogen’s rate of transmission to new hosts and its rate of killing its host (or of provoking the host’s immune system to eliminate it). Some biologists have criticized the tradeoff theory [2, 3], but others have successfully applied it to a variety of host-pathogen systems [4–7].

Fraser *et al.* have showed that HIV appears to satisfy the prerequisites of the tradeoff theory. The *set-point viral load* (SPVL: i.e., the characteristic virus load measured in blood during the intermediate stage of infection) is a measurable proxy for the rate of HIV within-host reproduction. Higher viral loads are correlated with faster progression to AIDS (higher virulence). Studies of discordant partnerships—stable sexual partnerships with one infected and one uninfected partner—have shown that SPVL is (1) positively correlated with transmission (people with higher SPVL transmit HIV to their uninfected partners sooner) and (2) heritable (when the uninfected partner does become infected, their SPVL is similar to their originally infected partner’s). Furthermore, the rate of increase in transmission has a decreasing slope as progression time decreases, fulfilling the requirements of the tradeoff theory [8]. Subsequent studies [9–11] used these data to parameterize mechanistic models of HIV virulence evolution, suggesting that HIV invading a novel population would initially evolve increased virulence, peaking after approximately 100-200 years and then declining slightly to a long-term stable virulence level.

The work of Shirreff *et al.* [9], and particularly the predicted transient peak in HIV virulence midway through the epidemic, highlights the importance of interactions between epidemiological and evolutionary factors [12, 13]. However, despite these studies’ attention to detail at the individual or physiological level, the population-level contact structures used in these models are relatively simple.

Many existing models of HIV eco-evolutionary dynamics use implicit models that incorporate the average effects of within-couple sexual contact—without representing the explicit dynamics of partnership formation and dissolution or accounting for extra-pair contact; agent-based formulations are more realistic, but can make it difficult to tease apart the reasons behind particular epidemic phenomena. Here we explore the effects of incorporating explicit contact structure in eco-evolutionary models.

Because our main goal is to explore how conclusions about virulence evolution depend on the way in which contact structure is modeled, we consider a series of models with increasing levels of complexity in the contact structure, but simplify some of the other epidemiological processes (such as the within-host life history of HIV). We evaluate our models across a wide range of parameters, using a Latin hypercube design; for each model run, we compute a set of metrics that summarize the evolutionary trajectory of SPVL over the course of the epidemic.

## Materials and methods

### Infection dynamics

Our models explicitly track the evolution of the distribution of log_10_ SPVL (which we denote as *α*) rather than the rate of progression to AIDS itself (hereafter we use “virulence” to denote log_10_ SPVL). We use a single-stage model of HIV that assumes constant infectivity over the course of an exponentially distributed infectious period. This assumption contrasts with Shirreff *et al.*’s previous model which explicitly tracked three stages of HIV infection (primary, asymptomatic, and AIDS) and used a more realistic Weibull-distributed infectious period. We show below that our results are not overly sensitive to this simplification, although it could conceivably affect our conclusions about the evolution of virulence (e.g., Kretzschmar and Dietz [14] show that pair formation dynamics and multiple stages of infectivity have interactive effects on $R_{0}$). In Shirreff *et al.*’s model, the transmission rate and infection duration depend on virus load only during the asymptomatic stage. In order to adapt their parameterization to our single-stage model, we used their parameters to compute the SPVL-dependent transmission rate and duration during the asymptomatic stage and then derived the overall duration of the infectious period as the sum of the three stage durations and the average transmission rate as the duration-weighted average of the three stage-specific transmission rates. Thus the within-couple transmission rate, *β* (see “Contact Structure” below), for our models is given by:

$$\beta \left(\alpha \right)=\frac{D_{P}\beta_{P}+D_{A}\left(\alpha \right)\beta_{A}\left(\alpha \right)+D_{D}\beta_{D}}{D_{P}+D_{A}\left(\alpha \right)+D_{D}},$$ (1)

where the duration of infection (*D*_*P*_ and *D*_*D*_) and rate of transmission (*β*_*P*_ and *β*_*D*_) of the Primary and Disease stages of infection are independent of the host’s SPVL (Table 1 gives definitions, units, and values for all parameters). Following Shirreff *et al.*, the duration of infection (*D*_*A*_) and rate of transmission (*β*_*A*_) for the Asymptomatic stage are Hill functions of the SPVL:

$$\begin{array}{cc} D_{A}\left(\alpha \right) & =\frac{D_{\text{max}}D_{50}^{D_{k}}}{V{\left(\alpha \right)}^{D_{k}}+D_{50}^{D_{k}}},\\ \beta_{A}\left(\alpha \right) & =\frac{\beta_{\text{max}}V{\left(\alpha \right)}^{\beta_{k}}}{V{\left(\alpha \right)}^{\beta_{k}}+\beta_{50}^{\beta_{k}}}, \end{array}$$ (2)

where *V*(*α*) = 10^*α*^.

**Table 1:** Parameter ranges and values. Parameters with fixed values are kept constant throughout simulations while other parameter values are taken from Latin hypercube samples using ranges specified in the table. Values of *c* and *ρ* are doubled from those given by Champredon *et al.*[15] because we keep track of individuals in the model, while they keep track of couples. Starred (*) parameters (used in Fig 1), and descriptions of Hill function coefficients, are taken from [9].

Notation	Description	Range/Value	Source
*ρ*	Partnership formation rate	1/10-2/5 per year	[15]
*c*	Partnership dissolution rate	1/15-1/5 (1.25*) per year	[15]
*c*_*u*_/*c*_*w*_	Relative contact rate for uncoupled transmission	1/5-5	Assumption
*c*_*e*_/*c*_*w*_	Relative contact rate extra-couple	0.01-1	[15]
*β*_*P*_	Rate of transmission during primary infection	1.31-5.09 (2.76*) per year	[16]
*β*_*D*_	Rate of transmission during high transmission disease stage	0.413-1.28 (0.76*) per year	[16]
*D*_*P*_	Duration of primary infection	1.23/12-6/12 (0.25*) years	[16]
*D*_*D*_	Duration of high transmission disease stage	4.81/12-14/12 (0.75*) years	[16]
*β*_max_	Maximum rate of transmission during asymptomatic stage	0.317 per year	[9]
*β*_50_	SPVL at which infectiousness is half maximum	13938 copies per ml	[9]
*β*_*k*_	Hill coefficient: steepness of increase in infectiousness as a function of SPVL	1.02	[9]
*D*_max_	Duration of primary infection	25.4 years	[9]
*D*_50_	SPVL at which duration of asymptomatic infection is half maximum	3058 copies per ml	[9]
*D*_*k*_	Hill coefficient: steepness of decrease in duration as a function of SPVL	0.41	[9]
*σ*_*M*_	Mutation standard deviation of log_10_ SPVL	0.12	[9]
*α*_min_	Minimum log_10_ SPVL	2	[9]
*α*_max_	Maximum log_10_ SPVL	7	[9]
*n*	Number of strains	21 (51*)	Assumption
*μ*	Mean number of non-cohabiting sexual partners	0.103–1.206	[17]
*κ*	Squared coefficient of variation of number of non-cohabiting sexual partners	0.01–100	Assumption

In models that allow extra-pair contact, the uncoupled and extra-couple transmission rates (i.e., the rates of transmission among people outside of a stable partnership, or between people inside of a stable partnership and people other than their partner) are scaled by multiplying the within-couple transmission rate *β* by the contact ratios *c*_*u*_/*c*_*w*_ and *c*_*e*_/*c*_*w*_ (see S1 Appendix).

### Mutation

Over the course of infection, mutation occurs within the host. However, we follow Shirreff *et al.* in assuming that SPVL of the strain transmitted by an infected individual is determined by the SPVL at the time of infection and is not further affected by within-host mutation. Instead, the mutational effect is modeled as occurring in a single step at the time of transmission.

First, the distribution of log_10_ SPVL is discretized into a vector:

$$\alpha_{i}=\alpha_{\text{min}}+\left(\alpha_{\text{max}}-\alpha_{\text{min}}\right)\frac{i-1}{n-1}\;i=1,2,3,\ldots n.$$ (3)

**Fig 1:**
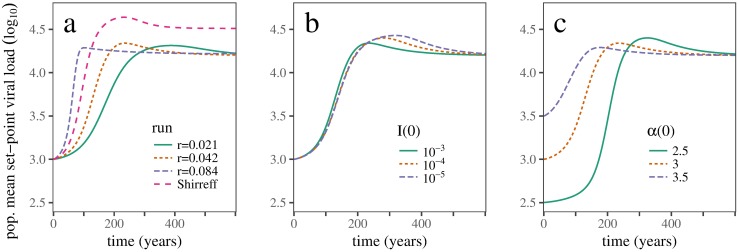
Baseline dynamics. Time series of mean population log_10_ SPVL using baseline parameters. (a) Contrast between the three-stage Shirreff model and the single-stage model calibrated to varying initial exponential growth rates, *r*. (b) Effects of varying initial infectious density *I*(0). (c) Effects of varying initial mean virulence *α*(0). The middle *r* = 0.042 (orange, dotted) curve in panel (a), calibrated to match the epidemic dynamics of Shirreff *et al.*’s three-stage model [9], shows that our simplified single-stage HIV model can produce similar SPVL trajectories to the original three-stage model; the other *r* values are chosen to show the effects of doubling or halving the initial epidemic growth rate. The other panels (b,c) show that eco-evolutionary dynamics are qualitatively similar across a range of initial conditions.

We have experimented with varying degrees of discretization in the strain distribution (i.e., values of *n*); in our model runs comparing results with Shirreff *et al.* [9] (Fig 1) we use *n* = 51 (i.e. a bin width of 0.1 log_10_ SPVL for *α*), but reducing *n* to 21 (bin width = 0.25 log_10_ SPVL) makes little difference; we use this coarser grid for all other simulations reported.

We then construct an *n* × *n* mutational matrix, *M*—which is multiplied with the transmission term—so that *M*_*ij*_ is the probability that a newly infected individual will have log_10_ SPVL of *α*_*j*_ given that their infected partner has log_10_ SPVL of *α*_*i*_. Finally, the probabilities are normalized so that each row sums to 1:

$$M_{ij}=\frac{\Phi \left(\alpha_{j}+d/2;i\right)-\Phi \left(\alpha_{j}-d/2;i\right)}{\Phi \left(\alpha_{\text{max}}+d/2;i\right)-\Phi \left(\alpha_{\text{min}}-d/2;i\right)},$$ (4)

where Φ(*x*;*i*) is the Gaussian cumulative distribution function with mean *α*_*i*_ and variance of $\sigma_{M}^{2}$, and *d* = (*α*_max_ − *α*_min_)/(*n* − 1). Unlike Shirreff *et al.*, who allowed for variation in the expressed phenotype (SPVL) of each genotype, we use a one-to-one genotype-phenotype map. Thus there is a single value for within-couple transmission rate and for progression rate corresponding to each SPVL compartment in the model:

$$\begin{array}{cc} \beta_{i} & =\beta (\alpha_{i}),\\ \lambda_{i} & =\frac{1}{D_{P}+D_{A}\left(\alpha_{i}\right)+D_{D}}. \end{array}$$ (5)

### Contact structure and partnership dynamics

We developed seven multi-strain evolutionary models covering a gamut including Champredon *et al.*’s relatively realistic [15] and Shirreff *et al.*’s relatively simple [9] contact structures, each of which is based on different assumptions regarding contact structure and partnership dynamics. Specifically, we focus on the effects of the assumptions of (1) instantaneous vs. non-instantaneous partnership formation; (2) zero vs. positive extra-pair sexual contact and transmission; and (3) homogeneous vs. heterogeneous levels of sexual activity on the evolution of mean log_10_ SPVL.

**Fig 2:**
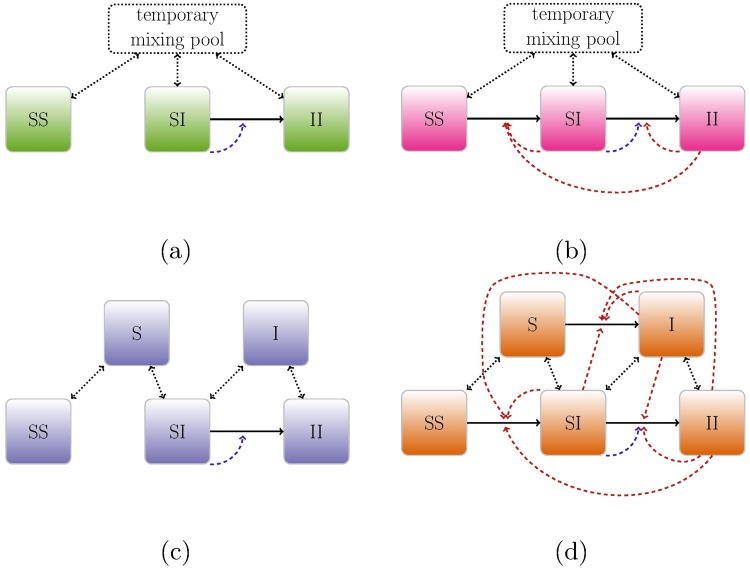
Model contact structures. Schematic representations of models with explicit contact structure. Top row (a,b) shows models with instantaneous partnership formation; bottom row (c,d) shows those where individuals remain single for some period of time between partnerships. Left column (a,c) shows models with within-partnership transmission only; in these models transmission can only occur within serodiscordant partnerships, and serodiscordant partnerships can only be formed by dissolution and reformation of partnerships. Right column (b,d) shows models that allow extra-pair and uncoupled transmission. *Solid arrows* represent infection transitions; *dotted arrows* represent partnership dissolution and formation; *dashed arrows* show influences on infection rate, including both within-pair transmission (blue) and extra-pair/uncoupled transmission (red). In addition to these models, we also use random-mixing and implicit models with only *S* and *I* compartments and heterogeneous models where each compartment is subdivided by sexual activity rate (of both partners, in the case of partnership compartments).

Our first four models (Fig 2) explicitly consider partnership dynamics [15]. The first (Fig 2d) assumes non-instantaneous partnership formation (i.e. individuals spend some time uncoupled, outside of partnerships) and consists of five states that are classified by infection status and partnership status; single (uncoupled) susceptible individuals (*S*), single infected individuals (*I*), concordant negative (susceptible-susceptible) couples (*SS*), discordant (susceptible-infected) couples (*SI*), and concordant positive (infected-infected) couples (*II*). The rates of pair formation are based on the numbers of uncoupled susceptible and infected individuals and the pair-formation rate; partnerships can either dissolve into singletons or be transformed into other types of partnerships by infection of one partner. This model also includes extra-pair contact with both uncoupled individuals and individuals in other partnerships (we denote it “pairform+epc”), so that susceptible uncoupled individuals and susceptible partners in any type of partnership can be infected by infected uncoupled individuals or infected partners in any type of partnership.

Specifically, single individuals (*S* and *I*) form partnerships at a *per capita* rate *ρ*, and partnerships dissolve at a rate *c*. Infected individuals in a discordant partnership infect their susceptible partner at a rate *β* (within-couple transmission rate) and susceptible individuals outside the partnership at a rate *c*_*e*_ (extra-couple transmission rate). Infected individuals in seropositive (*II*) partnerships can also infect any susceptible individual at rate *c*_*e*_. Likewise, single infected individuals (*I*) can infect any susceptible individuals (single individuals *S*, or susceptible members of *SS* or *SI* partnerships) at a rate *c*_*u*_ through uncoupled mixing. This parameterization follows Champredon *et al.*; we have adapted some of the details of their model to a multi-strain scenario, so that we track (for example) a matrix *II*_*ij*_ that records the number of concordant, HIV-positive partnerships in which the two partners have log_10_ SPVL of *α*_*i*_ and *α*_*j*_.

Our second model (“pairform”, Fig 2c) only considers within-couple transmission, in which case infection can only occur within a serodiscordant partnership; that is, we set *c*_*e*_ and *c*_*u*_ to zero.

Our third and fourth models, which are intended to bridge the gap between models with fully explicit pair-formation dynamics and the simpler, implicit models used by Shirreff *et al.* [9], assume instantaneous partnership formation (“instswitch”). The compartmental structure thus omits the single states *S* and *I*, comprising only the three partnered states: *SS*, *SI*, and *II*. Like the first two models, this pair of models differs in their inclusion of extra-pair contact: the third model (“instswitch+epc”, Fig 2b) includes extra-pair contact (now only with individuals in other partnerships, since uncoupled individuals do not exist in this model) while the fourth (“instswitch”, Fig 2a) only considers within-couple transmission. Although these models can also be implemented by setting the partnership formation rate of the explicit partnership models to a high value (we have tested that both methods in fact produce same results), we model instantaneous partnership formation models independently so that scaling the partnership formation rate during model calibration (see *Simulation runs* below) does not affect the eco-evolutionary dynamics.

The fifth and sixth models represent extreme simplifications of sexual partnership dynamics. The fifth (“implicit”) is an implicit serial monogamy model based on the epidemiological model used by Shirreff *et al.* [9]. It is a random-mixing model that explicitly tracks only the total number of susceptible and infected individuals. However, to reflect the effect of partnership structure, it uses an adjusted transmission rate derived from an approximation of the basic reproduction number of a serial monogamy model with instantaneous pair formation [16]. The sixth model (“random”) is a simple random-mixing model.

Lastly, we incorporated heterogeneity in sexual activity into the models. Individuals are divided into different risk groups based on their level of sexual activity; we scale all aspects of sexual activity, assuming that sexual activity level in both within- and extra-couple contacts is directly proportional to number of non-cohabiting (extra-couple and uncoupled) partners per year [17] (see S1 Appendix for full model details). We assume random activity-weighted mixing between risk groups [18]. (In the main text we focus on the model with non-instantaneous pair formation, extra-pair contact (“pairform+epc”) and heterogeneous sexual activity, which we denote as “hetero”; Fig D in S2 Appendix presents results on the effect of adding heterogeneity to other model variants.) While this model lacks some important elements, such as age-structured mixing patterns, needed for realistic models of HIV transmission in sub-Saharan Africa, it represents a first step toward assessing the effects of epidemiological complexity. As even the models shown here push the limits of compartmental-based models (the heterogeneity model comprises 24530 coupled ordinary differential equations), adding further complexity will probably require a shift to an agent-based model framework, as well as considerable effort in model calibration [10, 19, 20].

For simplicity (and following Shirreff *et al.*), all of our base models use an SIS (susceptible-infected-susceptible) formulation, where there is no natural mortality (and no explicit introduction of newly sexually active individuals into the susceptible pool). Individuals who die from AIDS are immediately replaced by an individual in the uncoupled-susceptible compartment. While admittedly unrealistic, this approach is reasonable given that (1) the natural mortality rate is low relative to the epidemiological dynamics and (2) the infectious period is long, so that the overall rates of disease-induced mortality and recruitment of newly sexually active individuals would roughly balance. To check the importance of this assumption, we also built models with vital dynamics where individuals dying from AIDS are removed from the population, with constant recruitment rates and constant low *per capita* natural mortality rates; this additional structure had only minor effects on the results.

### Initial conditions

Choosing the initial conditions for the simulations is challenging. In many modeling studies, researchers are primarily interested in equilibria (or other long-term dynamical attractors such as limit cycles) and are exploring models that have a single stable attractor, so initial conditions can be ignored as long as we run models for long enough. In eco-evolutionary dynamics, however, the initial conditions do affect our conclusions. We have no empirical information that would justify a particular choice of the fraction infected and the mean and variance of the distribution of SPVL at the point when the pandemic strain of HIV-1 entered the human population; in any case, the level of realism of our model does not support such a detailed consideration of the early dynamics of HIV. In most cases, we started with an initial log_10_ SPVL of 3.0, to match the value used by Shirreff *et al.* [9]. Shirreff *et al.* use an initial prevalence *I*(0) = 10^−3^; because we calibrated parameters based on the initial epidemic growth rate (see “Simulation runs” below), we set *I*(0) to 10^−4^ for most runs to ensure that the exponential growth phase lasted long enough for reliable estimation of the initial growth rate.

S1 Appendix provides further details on initial conditions, such as the initial distribution of SPVL around the mean and the distribution of initial infected density across single people and different partnership types.

### Sensitivity analyses

We ran most of our models across a wide range of parameters, as described in the next (*Latin hypercube sampling*) section. In several cases, however, we inspected only a few parameter sets, to qualitatively assess the sensitivity of the models to initial conditions or to model structure. In particular, we tested model sensitivity to the initial prevalence, *I*(0), and initial mean log_10_ SPVL, *α*(0), using baseline values of all parameters (Table 1). Using baseline parameter values, we also ran all four basic model structures (Fig 2) with vital dynamics and with heterogeneity in sexual contact, to assess the sensitivity of our results to these phenomena.

### Latin hypercube sampling

Despite considerable effort [15, 16], the parameters determining the rate and structure of sexual partnership change and contact are still very uncertain; this uncertainty led Champredon *et al.* [15] to adopt a Latin hypercube sampling (LHS) strategy [21] that evaluates model outcomes over a range of parameter values. In order to make sure that our comparisons among models apply across the entire space of reasonable parameter values, and in order to evaluate the differential sensitivity of different model structures to parameter values, we follow a similar protocol and perform LHS over a parameter set including both the early- and late-stage transmission and duration parameters (*β*_*P*_, *D*_*P*_, *β*_*D*_, *D*_*D*_) and contact/partnership parameters (*ρ*, *c*, *c*_*u*_/*c*_*w*_, and *c*_*e*_/*c*_*w*_). For the heterogeneity model, the mean and squared coefficient of variation (CV) for the number of non-cohabiting partners are sampled as well. We do not allow for uncertainty in parameters that are directly related to the evolutionary process (*β*_max_, *β*_50_, *β*_*k*_, *D*_max_, *D*_50_, *D*_*k*_, *σ*_*M*_), instead using Shirreff *et al.*’s point estimates throughout [9].

Latin hypercube sampling is done as in Champredon *et al.* [15]. For each parameter, *z*, its range is divided into *N* = 1000 equal intervals on a log scale:

$$z_{i}=\exp\left(\log\left(z_{\text{min}}\right)+\left[\log\left(z_{\text{max}}\right)-\log\left(z_{\text{min}}\right)\right]\frac{i-1}{N-1}\right)\;i=1,2,3,\ldots ,N.$$ (6)

Random permutations of these vectors form columns in a sample parameter matrix; each row contains a different parameter set that is used for one simulation run.

Table 1 gives the ranges of the model parameters used for LHS. Ranges of parameters controlling contact and partnership dynamics (*ρ*, *c*, and *c*_*e*_/*c*_*w*_) are taken from Champredon *et al.* [15], whereas those controlling infection (*β*_*P*_, *D*_*P*_, *β*_*D*_, and *D*_*D*_) are taken from Hollingsworth *et al.* [16]. The remaining parameter values are taken from Shirreff *et al.* [9].

One new parameter in our model, the ratio of uncoupled to within-couple transmission *c*_*u*_/*c*_*w*_, is needed to more flexibly contrast uncoupled and extra-couple transmission dynamics within multi-strain models (see S1 Appendix). Since it appears neither in either Shirreff *et al.* nor Champredon *et al.*’s models, we need to pick a reasonable range for it. Champredon *et al.* [15] assume that the effective within-couple contact rate and effective uncoupled contact rate have the same range of 0.05—0.25. Given Champredon *et al.*’s parameter range, the possible maximum and minimum values of *c*_*u*_/*c*_*w*_ are 5 and 1/5. Therefore, we use 1/5-5 as the range for the parameter *c*_*u*_/*c*_*w*_. Although this adds more uncertainty to the parameter *c*_*u*_—Champredon *et al.*’s range implies a 5-fold difference whereas ours gives a 25-fold difference—we consider the wider range appropriate, as little is known about the uncoupled transmission rate.

Two parameters, mean and the squared coefficient of variation (CV) of number of non-cohabiting partners, are sampled for heterogeneity in sexual activity. To allow for a wide range of uncertainty, range for the mean number of non-cohabiting partners was taken from unmarried men, as that was the group with the largest variability [17]. Omori *et al.* [17] give a very wide range for the coefficient of variation (≈ 0—20, corresponding to squared CV range of 0-400): we narrowed this range for CV^2^ to 0.01-100. At the bottom end of the range, an observation that a group behaves perfectly homogeneously (*CV* = 0) is likely to be a sampling artifact; at the upper end, the estimate is also likely to be noisy because of the low mean value among married females (who have the largest range of CV). We assume that the number of non-cohabiting partners follows a Gamma distribution.

### Simulation runs

One of the hardest parts of model comparison is finding parameter sets that are commensurate across radically different model structures. For the most part, our models are too complex to derive analytical correspondences among the parameters for different models. Given a numerical criterion, such as *r* (initial exponential growth rate) or $R_{0}$ (intrinsic reproductive number), we can adjust one or more parameters by brute force to ensure that all of the models match according to that criterion. While $R_{0}$ is often considered the most fundamental property of an epidemic, and might thus seem to be a natural matching criterion, here we focus on matching the initial growth rate *r* for several reasons. First, our primary interest is in the transient evolutionary dynamics of virulence, which are more strongly affected by *r* than $R_{0}$. Second, *r* is more directly observable in real epidemics; *r* can be estimated by fitting an exponential curve to the initial incidence or prevalence curves [22], while $R_{0}$ typically requires either (1) knowledge of *all* epidemic parameters or (2) calculations based on *r* and knowledge of the serial interval or generation interval of the disease [23]. Thus, we scale parameters so that every run has the same initial exponential growth rate in disease prevalence.

In order to allow for all models to have equal initial exponential growth rate, *r*, we need to pick a parameter, *s*, such that lim_*s*→0_ *r*(*s*) = 0 and lim_*s*→∞_ *r*(*s*) = ∞. As adjusting either partnership change rate (i.e. partnership formation and dissolution rate) or transmission rate fails this requirement for some of our models, we scaled both partnership change rate and transmission rate by the same factor *γ*: *β*_adj_ = *γ* *β*_base_, *c*_adj_ = *γ* *c*_base_, *ρ*_adj_ = *γ* *ρ*_base_. Since transmission rate is scaled by *γ*, uncoupled and extra-couple transmission rates are adjusted as well. For the instantaneous-switching and implicit models, none of which track single individuals, only the transmission rate and partnership dissolution rate (in this case equivalent to the partnership change rate) are adjusted.

We run each model for each of 1000 parameter sets chosen by Latin hypercube sampling, with fixed starting conditions of mean log_10_ SPVL of 3.0, standard deviation of log_10_ SPVL of 0.2, and epidemic size of 10^−4^. After each run, the initial exponential growth rate is calculated. Then the parameters are scaled as described above so that the initial exponential growth rate is scaled to 0.04 year^−1^, a value that approximates the growth rates of Shirreff *et al.*’s original models. When calibrating, we run each model for only 500 years (full simulations are run for 4000 years), which is always long enough to capture the exponential growth phase of the model. We use a 4/5 order Runge-Kutta method (ode45 from the deSolve package [24]) for all simulations. (For the heterogeneous model, approximately 10% of the samples failed due to numerical instability; we discarded these runs.)

For each model we derive the following summary statistics: maximum population mean log_10_ SPVL; time at which this maximum occurs (corresponding to peak virulence—this is also the time at which the maximum rate of progression and maximum transmission rate occur); equilibrium log_10_ SPVL; and minimum expected time to progression. Minimum expected progression time is obtained by applying the Hill function (eq 2) to the maximum mean log_10_ SPVL of each run. Equilibrium log_10_ SPVL is calculated after 4000 years of simulated time. Although most simulations reach equilibrium much earlier than 4000 years, we set this very long time horizon because a small subset of the simulation runs show very slow evolution rates.

Knowing the peak log_10_ SPVL, timing of the peak log_10_ SPVL/peak virulence, and equilibrium log_10_ SPVL provides sufficient detail to identify the overall shape of the virulence trajectory. In particular, knowing the timing of the peak virulence (how many years into the epidemic the virulence peaks) can help epidemiologists guess whether the virulence of an emerging pathogen is likely (1) to peak early, possibly even before the pathogen is detected spreading in the population, and decline over the remaining course of the epidemic; (2) to increase, peak, and decline over the foreseeable future; or (3) to increase very slowly, peaking only in the far future. To the extent that our simplistic model for HIV reflects reality, we would take the peak time of 150-300 years (Fig 1c) to mean that, in the absence of treatment, the epidemic would probably still be increasing in virulence.

## Results

Our simplifications of Shirreff *et al.*’s model [9] reproduce its qualitative behaviour—in particular, its predictions of virulence dynamics—reasonably well. As we calibrate the parameters to achieve initial epidemic growth rates *r* ranging from 0.042 year^−1^ to 0.084 year^−1^ (the former value matching the initial rate of increase in prevalence in Shirreff *et al.*’s full model) the initial trajectory of increasing virulence brackets the rate from the original model (Fig 1a). For matching initial growth rates (*r* = 0.042) the peak log_10_ SPVL occurs at the same time (≈ 200 years) but the peak virulence is lower than Shirreff’s (≈ 4.3 vs. ≈ 4.6 log_10_ SPVL), as is equilibrium virulence (≈ 4.25 vs. ≈ 4.5 log_10_ SPVL).

Changing the initial infectious density (*I*(0)) has little effect on the virulence trajectory. Decreasing *I*(0) makes SPVL peak slightly later and higher, because it allows a longer exponential-growth phase before the transition to endemic dynamics (Fig 1b). Decreasing the initial SPVL also leads to progressively later, higher peaks in SPVL (Fig 1c). In this case the delay in the peak is more pronounced than for low *I*(0), because the rate of SPVL increase is eventually limited by the mutation rate. The peak SPVL is actually larger for a lower starting SPVL, presumably because lower SPVL also allows for a longer epidemic phase. However, the peaks are similar across the entire range of initial conditions, because even in the most limited (high-*I*(0), high-*α*(0)) cases HIV can evolve close to its optimal growth-phase SPVL.

**Fig 3:**
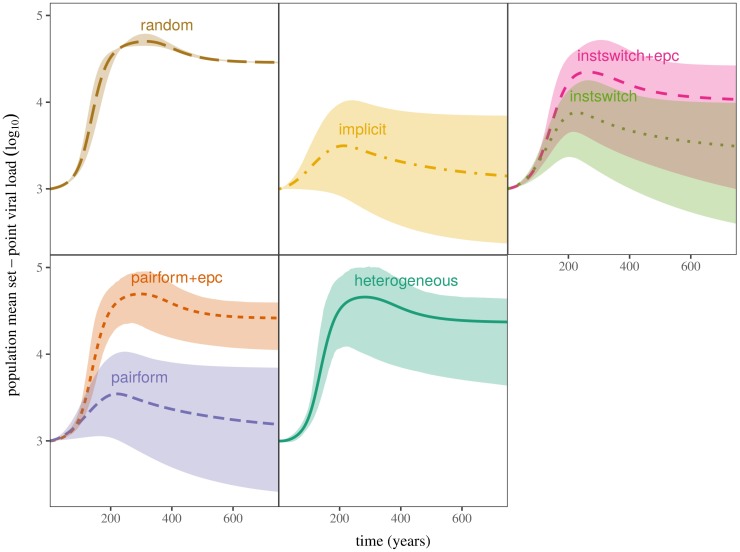
Envelopes of virulence trajectories (population mean log_10_ SPVL) under all models. Panels are arranged in order of complexity of contact structure, from “random” (least complex) to “hetero” (most complex). All models were run until *t* = 4000 years using parameters from 1000 Latin hypercube samples to illustrate the range of possible dynamics. Envelopes contain the middle 95% of trajectories (i.e. we select all points between the 0.025 and 0.975 quantiles for each model at each year), while center lines show mean trajectories. Truncated series (up to year 800) are shown here.

Across the entire range of parameters covered by the Latin hypercube samples, all of our models produce qualitatively similar virulence trajectories, which we quantify in terms of population mean log_10_ SPVL (Fig 3: higher population mean log_10_ SPVL corresponds to higher virulence). Although the speed of virulence evolution varies, leading to wide variation in the peak log_10_ SPVL (ranging from 3 to 5.5) because HIV can evolve farther toward its growth-phase optimum before the transition to the endemic phase, virulence peaks between 200 and 300 years in all models.

**Fig 4:**
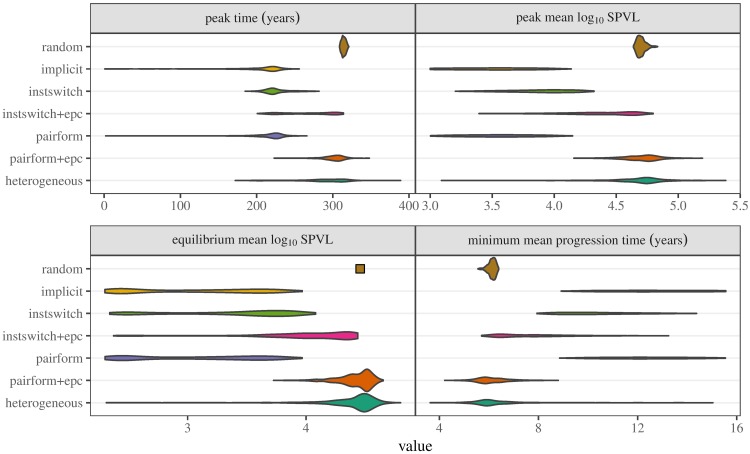
Univariate distributions of summary statistics. Models arranged in order of complexity, as in Fig 3. For every simulation, three summary statistics are shown: peak time, maximum mean log_10_ SPVL, and equilibrium mean log_10_ SPVL. Peak time is the time at which the SPVL reaches its maximum for each simulation run (maximum mean log_10_ SPVL). Equilibrium mean log_10_ SPVL is taken by taking the mean log_10_ SPVL value at *t* = 4000. Because the distribution of equilibrium mean log_10_ SPVL (lower left panel) for the random-mixing model is very narrow, it has been replaced by a point in order to preserve the vertical axis scaling.

**Fig 5:**
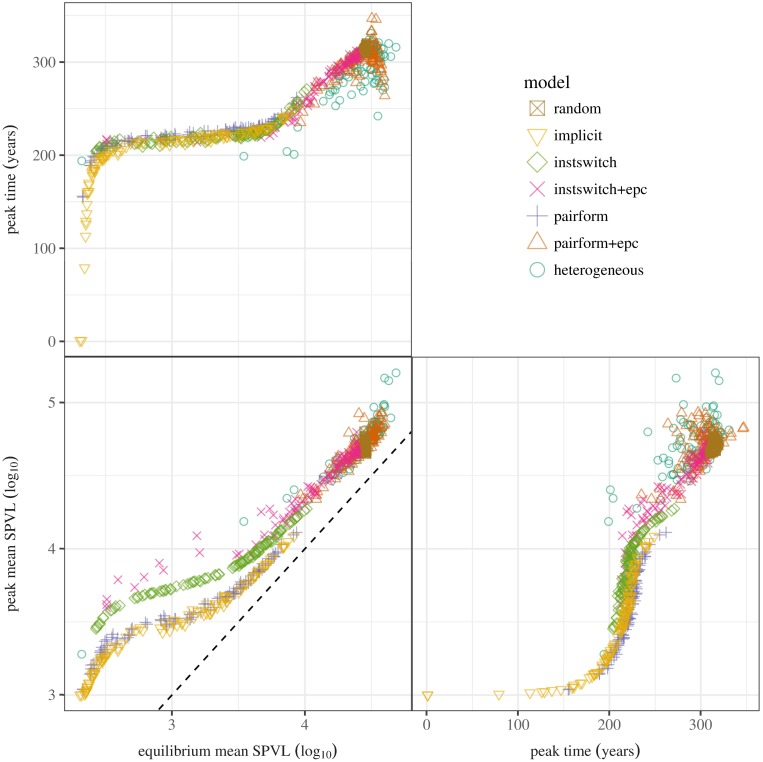
Pairs plot: bivariate relationships among summary statistics for each model structure. The three summary statistics for each simulation are plotted against each other in order to visualize the relationship among the summary statistics and to help compare models. Surprisingly, the implicit model, an approximation for instantaneous partnership formation model (instswitch), shows an almost identical trend with a model that has pair formation dynamics (pairform). The *dashed line* in the equilibrium vs. peak virulence plot (lower left) shows the 1:1 line, where equilibrium and peak virulence are equal. To avoid too much overplotting, only 10% of the parameter sets (randomly sampled) are shown here.

Our chosen summary statistics (peak time, maximum mean log_10_ SPVL, equilibrium mean log_10_ SPVL, and minimum mean progression time) all vary considerably across models (Fig 4). We first consider the models of intermediate realism: implicit, instantaneous-switching with and without extra-pair contact, and pair formation without extra-pair contact. Some parameter sets for these models lead to low equilibrium virulence (2.3-3 log_10_ SPVL). For these data sets, virulence may either increase from its initial value, reaching an early peak (≈ 200 years) between 3 and 4 log_10_ SPVL and then declining to a lower equilibrium value, or in extreme cases virulence may decline immediately, leading to a peak virulence (as we have defined it) equal to the starting value of *α*(0) = 3 log_10_ SPVL at *t* = 0 (Fig 5). At the opposite extreme, parameter sets that produce high equilibrium virulence (4.7 log_10_ SPVL) also produce late peaks (> 200 years) and high peak virulence (5.6 log_10_ SPVL).

**Fig 6:**
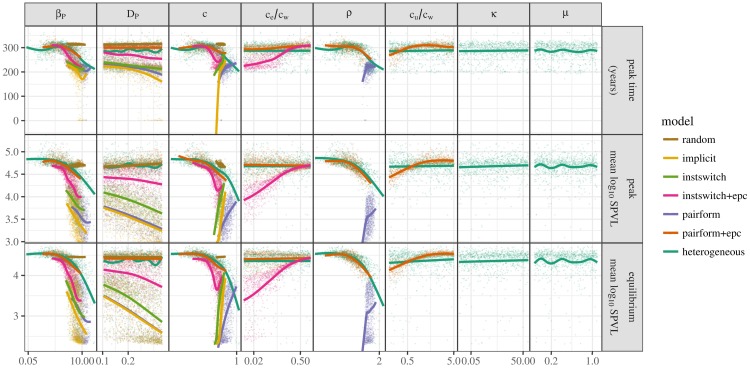
Sensitivity plot. Distribution (points) and trend (smooth line) of summary statistics (y-axis, rows) as a function of parameter values (x-axis, columns), some of which have been scaled to calibrate the initial epidemic growth rate. Changing parameters may have differing effects for models with different contact structures: for example, partnership dissolution rate (*c*, third column) increases peak time and virulence in models without explicit pair formation but decreases them in more realistic models.

The most striking aspect of the univariate comparisons in Fig 4 (and the bivariate comparisons in Fig 5), is the similarity between the results of the least (random-mixing) and the most complex (pair formation with extra-pair contact and pairform+epc with heterogeneity) models. The random-mixing model has the lowest variability, because it is unaffected by uncertainty in pair formation and extra-pair contact parameters, but otherwise the virulence dynamics of these three extreme models are remarkably similar. This phenomenon is driven by the strong effects of extra-pair contact in the model with explicit pair formation and extra-pair contact (“pairform+epc” in Figs 3–6). When individuals spend time uncoupled between partnerships, and when these single individuals can transmit disease to coupled individuals, the resulting unstructured mixing overwhelms the effect of structured mixing within partnerships, leading to mixing that is effectively close to random. Once unstructured mixing is strong, adding realistic heterogeneity of mixing to the model has little effect other than increasing the variability in the outcomes.

The random-mixing, pairform+epc, and heterogeneous models all predict high population mean log_10_ SPVL at the virulence peak (median (95% CI) = 4.7 (4.65-4.79), 4.72 (4.37-4.96), 4.72 (4.09-5.03)). In contrast, the implicit model predicts a much lower peak log_10_ SPVL value: 3.52 (3-4.02) years. The random-mixing, pairform+epc, and heterogeneous models predict rapid progression to AIDS at the virulence peak (median/95% CI = 6.1 (5.7-6.3), 6.02 (5.04-7.7), 6.03 (4.8-9.2)), while the implicit model predicts minimum progression times about twice as long (12.5 (9.6-15.6) years). The corresponding differences in mean within-couple transmission probability at the peak are even more extreme, about a fourfold difference: 0.249 (0.24-0.26), 0.252 (0.19-0.28), and 0.252 (0.15-0.28) per year for the random and pairform+epc models vs. 0.059 (0.02-0.13) per year for the implicit model. (S2 Appendix presents plots showing univariate summaries of expected progression time to AIDS and transmission probability.)

Bivariate relationships (Fig 5) help distinguish the results of different models with similar univariate distributions of dynamical summaries. While the relationship between equilibrium log_10_ SPVL and peak time is similar for all model structures (top left panel), the other relationships show more variation. In particular, the implicit and pair-formation (without extra-pair contact) models are very similar to each other, but distinct from the other models. We still do not have a convincing explanation for this distinction; we would have expected the implicit model to be most similar to the the instantaneous-switching model without extra-pair contact, which most closely matches its underlying assumptions. However, we note that the implicit model derivation is based on defining the force of infection to match a scaled version of $R_{0}$, and as such would be expected to match the equilibrium behaviour but not necessarily the epidemic-phase behaviour of a model with explicit partnership dynamics.

Finally, the sensitivity plot (Fig 6) shows the effects of each parameter on the summary statistics. The most notable difference can be observed by comparing the scaled parameters (e.g. *β*_*P*_, *β*_*D*_, *c*, *ρ*) with the unscaled parameters (e.g. *D*_*P*_, *D*_*D*_, *c*_*e*_/*c*_*w*_, *c*_*u*_/*c*_*w*_, *κ*, *μ*); the effects of *β*_*D*_ and *D*_*D*_ are not shown in Fig 6 as they show patterns almost identical to *β*_*P*_ and *D*_*P*_, respectively.

For the scaled parameters, the parameter ranges (horizontal axis) are compressed for models without extra-pair contact because these models require a large amount of parameter scaling in order to achieve the specified initial epidemic growth rate (*r* = 0.04). In contrast, models with extra-pair contact show a wide range of parameters as they can display a wide range of dynamics depending on *c*_*e*_/*c*_*w*_ (as well as *c*_*u*_/*c*_*w*_ for models with uncoupled mixing) and thus require a wide range of scaling factors to achieve the target growth rate. Parameter ranges for the random-mixing model (especially *c*) are severely compressed because this model has little flexibility.

For parameters involved in partnership turnover (*c* and *ρ*), the figure again shows differences between models with and without extra-pair contact. Models with extra-pair contact show a gradual decrease in peak time, maximum log_10_ SPVL, and equilibrium log_10_ SPVL with increasing turnover rates. Increases in the other parameters lead to increases in all three summary statistics. In these models, increased turnover rates diminish the effect of extra-pair contact, thus selecting for lower log_10_ SPVL. For models without extra-pair contact, increased turnover rates decrease the level of structured mixing (mimicking extra-pair contact models), resulting in selection for higher log_10_ SPVL.

The implicit model and the instantaneous partnership formation model show similar patterns in scaled parameters. In fact, the effect of partnership dissolution rate, *c*, on equilibrium log_10_ SPVL is almost identical in these models (although they can be distinguished in Fig 5). Lastly, increasing in transmission rates (*β*_*P*_ and *β*_*D*_) causes the summary statistics to decrease in all models except the random-mixing model.

Surprisingly, once calibration is taken into account, the unscaled parameters have little effect overall. Increase in duration (*D*_*P*_, *D*_*D*_) in the primary and disease stages generally decreases the equilibrium virulence, peak virulence, and peak time, although the models with uncoupled mixing and random-mixing model have high, relatively constant values with respect to these parameters. The ratio of extra-pair to within-pair contact (*c*_*e*_/*c*_*w*_) affects summary statistics in the instantaneous-switching +epc model, but not the pair-formation+epc model (probably because the uncoupled individuals present in the pair-formation+epc model make extra-pair contact by coupled individuals less important). Similarly, increasing the ratio of uncoupled to within-pair contact, *c*_*u*_/*c*_*w*_, increases peak and equilibrium log_10_ SPVL and delays peak time of the pair-formation+epc model but has almost no effect on the heterogeneous model. Neither the uncoupled contact rate nor the mean (*μ*) or CV^2^ of the number of non-cohabiting sexual partners has much systematic effect in the heterogeneous model.

Finally, incorporating additional realism to the model, i.e. combining heterogeneity with all four basic contact structures or allowing for vital dynamics rather than assuming an SIS model, leads to only small differences in the conclusions stated so far (Fig D in S2 Appendix). Relative to our baseline SIS assumption, the effect of adding vital dynamics is to delay the virulence peak slightly and increase both the peak and equilibrium virulence. The changes are small, however: across all models, the maximum increase in time until the virulence peak is 40 years (for the instswitch+epc model), in the peak log_10_ SPVL is 0.24 units (instswitch), and in the equilibrium log_10_ SPVL is 0.4 units (pairform). The changes in the most realistic model (pairform+epc) are considerably smaller: an increase of 2 years vs. a decrease of 13 years in the time to the virulence peak for the models with vital dynamics and heterogeneity, respectively; an increase of 0.1 units vs. a decrease of 0.01 units in peak log_10_ SPVL; and an increase of 0.145 units vs. a decrease of 0.03 units in equilibrium log_10_ SPVL. Thus, while we can never rule out the possibility of some higher-order interaction among epidemiological phenomena leading to significant changes in our conclusions, we are reasonably confident that the results reported here are robust to additional complexities.

## Discussion

How contact structures are modeled can strongly affect researchers’ conclusions about the evolutionary dynamics of virulence. In particular, a relatively simple, strategic eco-evolutionary model of HIV can predict peak log_10_ set-point viral loads (over the course of an epidemic) ranging from 3.5 to 4.8 depending on the specific model of sexual partnership behaviour used. This difference in log_10_ SPVL is epidemiologically significant, corresponding to a twofold difference (12 vs. 6 years) in expected time to progression.

The restriction of transmission within stable partnerships strongly limits eco-evolutionary dynamics by limiting the maximum speed of epidemic growth. An HIV genotype that optimizes SPVL to maximize the speed of spread in a homogeneous population will be sub-optimal in a context where infection can only spread beyond a partnership once it dissolves. This finding echoes a long line of studies that show that population structure leads to the evolution of “prudent” parasites, although most of these studies focus on equilibrium optima rather than eco-evolutionary dynamics [25–28]. The more complex contact structures we modeled mitigate these constraints by allowing HIV to spread among uncoupled individuals (through finite pair-formation) and members of stable partnerships (through extra-pair contact), albeit at lower rates than within partnerships.

Thus, we see the biggest differences not between the simplest and the most complex contact structures, which either ignore pair structure completely or allow for extra-pair contact that reduces its impact, but between the complex contact structures and models of *intermediate* complexity. These intermediate-complexity models attempt, quite reasonably, to add at least some of the realism of human sexual behaviour, but err by neglecting the apparently insignificant detail of extra-pair contact. If partial complexity may lead to such mistakes, how can modelers do anything but always strive to build the most realistic models possible?

All models must simplify the world. Many constraints—among them data availability, computation time, and code complexity—drive the need for parsimony, with different constraints applying in different contexts. The critical question that modelers must ask is whether the simplified model gives adequate answers, or whether the simplifications lead to qualitative or quantitative errors. This question is especially important for modelers who are hoping that their conclusions will guide management decisions.

In the particular example of HIV virulence eco-evolutionary dynamics and the complexity of contact structures we reach the slightly ironic conclusion that the effort put into building a more realistic model essentially cancels out, putting us back where we started when used a naive random-mixing contact model. However, we are not quite back where we started, as the complex models lead to wider, presumably more realistic confidence intervals on the predictions. In general, unstructured mixing—whether occurring through purely random mixing, or through extra-pair contact and contact among people outside of stable partnerships—tends to drive faster virulence evolution, leading to higher peak virulence and lower times to progression at the peak time.

Taking further steps to make the model even more realistic might add further structure, making the random-mixing model predictions less accurate. For example, our model forms partnerships randomly, and assumes that extra-pair contact is randomly mixing across the population; one could instead model extra-pair contact as arising from multiple concurrent partnerships (some, such as contact with sex workers, of very short duration) and/or more structured partnership formation (by age, ethnicity, or behaviour group). In contrast, the elevated viral load in the early stage of HIV infection, neglected in our model, will likely lead to higher maximum epidemic growth rates and allow more scope for transient viral evolution, although only if extra-pair contact is possible. The effects of other realistic complications such as explicit modeling of two sexes (both in contact structure and differential transmission probabilities), temporal and spatial variation in epidemic processes, or presence of genetic variation in hosts are harder to predict. As mentioned above, our compartmental model already requires tens of thousands of coupled differential equations, which will increase multiplicatively with additional model dimensions such as age, sex, or HIV stage. Thus, further model elaboration will best be done with agent-based models.

Parameterization is one of the biggest challenges of epidemiological modeling. In addition to following Champredon *et al.* [15] by doing Latin hypercube sampling across a wide range of epidemiological parameters, we calibrated each set of parameters to the same initial epidemic growth rate, chosen to match the results of previous models [9]. Previous models in this area have drawn their parameters from cohort studies from the 1990s [16, 29] rather than doing any explicit calibration to epidemic curves, but they give reasonable order-of-magnitude growth rates (≈ 0.04 year^−1^) for the early stages of the HIV epidemic (although considerably lower than estimates of ≈ 0.07 − 0.1 year^−1^ based on population genetic reconstructions [30]). However, our reason for calibrating was not to match any specific observed epidemic, but rather to make sure that we were making meaningful comparisons across a range of models with radically different contact structures, and hence involving different interpretations of the same quantitative parameters. For example, in models with instantaneous switching the partnership dissolution rate *c* is identical to the partnership formation rate; in models with explicit partnership formation, the partnership formation rate is also *c* at equilibrium, but might vary over the course of an epidemic. Models with equal parameters but different structures cannot be compared directly; calibration solves this problem.

More generally, any model that wants to be taken seriously for management and forecasting purposes should be calibrated to *all* available data, using informative priors to incorporate both realistic distributions of uncertainty for all parameters from independent measurements [31] and calibration from population-level observations of epidemic trajectories. Such a procedure would also be an improvement on the common—although not universal—practice, which we have followed here, of assessing uncertainty over uniform ranges rather than using distributions that allow more continuous variation in support over the range of a parameter.

Researchers have documented that HIV virulence and set-point viral load are changing, on time scales comparable to those portrayed here (e.g., compare Fig 3 to Herbeck *et al.*’s estimated rate of change of 1.3 log_10_ SPVL per century [95% CI -0.1 to 3] [32]), and have begun to build relatively realistic models that attempt to describe how interventions such as mass antiretroviral therapy (ART) can be expected to change the trajectory of virulence evolution [11, 33, 34]. While these efforts are well-intentioned, we caution that structural details that are currently omitted from these models could significantly change their conclusions.

## Supporting information

S1 Appendix Model details. (PDF)

S2 Appendix Dynamics of transmission and virulence. (PDF)
