# Supplementary material for: Effects of contact structure on the transient evolution of HIV virulence
Source: PLoS Comput Biol. 2017 Mar 31;13(3):e1005453. doi: 10.1371/journal.pcbi.1005453 (PMC5391972; doi:10.1371/journal.pcbi.1005453)
Supplement: S1 Appendix — (PDF) [file pcbi.1005453.s001.pdf]

Supporting Information S1: model details

Since we use multi-strain models in which the distribution of $\log_{10}$ SPVL has been discretized into a vector, we use a matrix notation to describe our models. The five states described in the *Methods* section are replaced with the following notations: S, I_i, SS, SI_i, II_{ij}, where the subscripts denote the strain with which an individual is infected. For example, I_i is number of infected individuals with $\log_{10}$ SPVL of α_i, and II_{ij} is the number of concordant, HIV-positive couples in which the two partners have $\log_{10}$ SPVL of α_i and α_j (independent of order; II_{ij} is synonymous with II_{ji}). Below, we use the Kronecker delta (i.e. $\delta_{ij} = 1$ if $i = j$ and 1 otherwise) in a slightly non-standard fashion as an exponent, e.g. $2^{\delta_{ij}}$, to set a value to 2 when $i = j$ and 1 otherwise.

Models 1 (“pairform+epc”) and 2 (“pairform”)

Partnership dynamics

Single individuals acquire partners at per-person rate ρ. Partnership formation rates for S and I_i are ρS and ρI_i, respectively. We follow Champredon *et al.* [1] in assuming that single individuals are distributed into coupled states with pair-formation (PF) rates as follows:

$$
\begin{aligned}
\mathrm{PF}(SS) &= \frac{\rho S \cdot S}{2(S + \sum_k I_k)}, \\
\mathrm{PF}(SI_i) &= \frac{\rho S \cdot I_i}{S + \sum_k I_k}, \\
\mathrm{PF}(II_{ij}) &= \left(\frac{1}{2}\right)^{\delta_{ij}} \cdot \frac{\rho I_i \cdot I_j}{S + \sum_k I_k}.
\end{aligned} \tag{1}
$$

Partnerships dissolve at per-partnership rate c: the dissolution rates for SS, SI_i, and II_{ij} pairs are cSS, cSI_i, and cII_{ij} respectively. Unlike a single-strain model, where both individuals leaving the II partnership would enter I, we have to account for strains with which the individuals in concordant partnership are infected (i.e. both partners in II_{ii} enter I_i whereas one partner in II_{ij} enters the I_i compartment while the other enters I_j). Thus, coupled individuals are distributed into single states through partnership dissolution (DS) rates:

$$\begin{aligned} \mathrm{DS}(S) &= 2cSS + \sum_k cSI_k, \\ \mathrm{DS}(I_i) &= cSI_i + \sum_k 2^{\delta_{ik}} cII_{ik}. \end{aligned} \tag{2}$$

Combining the partnership formation and dissolution processes yields the following equation:

$$\begin{aligned} S' &= -\rho S + 2cSS + \sum_k cSI_k \\ I_i' &= -\rho I_i + cSI_i + \sum_k 2^{\delta_{ik}} cII_{ik} \\ SS' &= \frac{\rho S \cdot S}{2(S + \sum_k I_k)} - cSS \\ SI_i' &= \frac{\rho S \cdot I_i}{S + \sum_k I_k} - cSI_i \\ II_{ij}' &= \left(\frac{1}{2}\right)^{\delta_{ij}} \cdot \frac{\rho I_i \cdot I_j}{S + \sum_k I_k} - cII_{ij} \end{aligned} \tag{3}$$

Pair-formation models: infection dynamics

Within-couple transmission (WT) occurs in both models. An infected partner in SI partnership transmits virus to a susceptible partner at per-partnership rate β: $\mathrm{WT}(SI_i) = -\beta_i SI_i$. Since we assume that mutation occurs, II_{ij} pairs, where $i \neq j$, can be formed from either SI_i or SI_j partnerships: $\mathrm{WT}(II_{ij}) = M_{ij}\beta_i SI_i + M_{ji}\beta_j SI_j$. On the other hand, II_{ii} can only be formed from an SI_i partnership: $\mathrm{WT}(II_{ii}) = M_{ii}\beta_i SI_i$. Using the Kronecker delta notation, we obtain the following set of equations for within-couple transmission dynamics:

$$\begin{aligned} \mathrm{WT}(SI_i) &= -\beta_i SI_i, \\ \mathrm{WT}(II_{ij}) &= \left(\frac{1}{2}\right)^{\delta_{ij}} \cdot (M_{ij}\beta_i SI_i + M_{ji}.\beta_j SI_j) \end{aligned} \tag{4}$$

Champredon *et al.* [1] define the proportion of infectious extra-couple and uncoupled contact through the following term:

$$P = \frac{c_u I + c_e(SI + 2II)}{c_u(S + I) + 2c_e(SS + SI + II)}. \tag{5}$$

The effective uncoupled, c_u, and extra couple, c_e, contact rates are the product of uncoupled/extra-

couple contact rate $\times$ rate of transmission per contact. Therefore, the transmission rate per contact term in c_u and c_e is canceled out in the equation above. Using this property, we modify the equation above as follows:

$$P = \frac{r_u I + r_e(SI + 2II)}{r_u(S + I) + 2r_e(SS + SI + II)}, \tag{6}$$

where $r_u = c_u/c_w$ and $r_e = c_e/c_w$ are the relative uncoupled/extra-couple contact rates. This simplification is useful in a multi-strain model since we cannot multiply a vector by a scalar value (e.g. $c_u S$ in denominator) if we use Champredon *et al.*'s equation in its original form. Extending the above equation to the multi-strain model so that P_i represents the proportion of the extra-couple and uncoupled contact of an infected individual with strain i, we obtain:

$$P_i = \frac{r_u I_i + r_e(SI_i + \sum_k (II_{ik} + \delta_{ik} II_{ik}))}{r_u(S + \sum_k I_k) + r_e(2SS + \sum_k 2SI_k + \sum_l \sum_k 2^{\delta}_{lk} II_{lk})}. \tag{7}$$

Using the equation above, we can model extra-pair transmission (ET). For convenience, uncoupled and extra-couple transmission rates, c_u and c_e, will be replaced with $U_i = r_u \beta_i$ and $E_i = r_e \beta_i$ hereafter.

Single susceptible individuals become infected through uncoupled contact at per-person rate $\sum_k P_k U_k$ and enter the single infected state. Through mutation, newly infected individuals are distributed into single infected compartments with different strains: $\text{ET}(I_i) = \sum_k M_{ki} P_k U_k S$. Either partner in an SS partnership can become infected at per-person rate $\sum_k P_k E_k$, and partnership state changes to an SI partnership at the total rate of $\sum_i 2P_i E_i SS$. The formation of SI_i partnerships is similar to the process through which single susceptible individuals are distributed into single infected compartments: $\text{ET}(SI_i) = \sum_k 2M_{ki} P_k E_k SS$. Lastly, the susceptible partner in an SI partnership can become infected from extra-couple contacts at a per-person rate of $\sum_k P_k E_k$, so that the partnership changes to an II partnership. As in the previous cases, SI_i partnerships are lost at a rate of $\sum_k P_k E_k SI_i$. The mutation process is similar to that of within-couple transmission. The only difference is that the $\log_{10}$ SPVL of a newly infected partner is not determined by its social partner but from an extra-couple partner (i.e. the term P_i): $\text{ET}(II_{ij}) = (\frac{1}{2})^{\delta_{ij}} (\sum_k (M_{kj} P_k E_k SI_i + M_{ki} P_k E_k SI_j))$. Combining these equations we get the following set of equations that describe the complete transmission dynamics:

$$
\begin{aligned}
S' &= -\sum_k P_k U_k S, \\
I_i' &= \sum_k M_{ki} P_k U_k S, \\
SS' &= -\sum_i 2P_i E_i SS, \\
SI_i' &= \sum_k 2M_{ki} P_k E_k SS - \beta_i SI_i - \sum_k P_k E_k SI_i, \\
II_{ij}' &= \left(\frac{1}{2}\right)^{\delta_{ij}} \cdot (M_{ij}\beta_i SI_i + M_{ji}, \beta_j SI_j) + \left(\frac{1}{2}\right)^{\delta_{ij}} \cdot (\sum_k (M_{kj} P_k E_k SI_i \\
&\quad + M_{ki} P_k E_k SI_j)).
\end{aligned} \tag{8}
$$

Pair formation models: Disease induced mortality

The per-person disease induced mortality (DM) rate, λ, is given by taking the reciprocal of the total duration of the infection: $\lambda_i = 1/(D_A + D_P(\alpha_i) + D_D)$. Since we assume an SIS formulation, where infected individuals that die from infection are immediately replaced by an individual in the single susceptible compartment, we obtain the following equation for single infected individuals:

$$
\begin{aligned}
\text{DM}(S) &= \sum_k \lambda_k I_k, \\
\text{DM}(I_i) &= -\lambda_i I_i.
\end{aligned} \tag{9}
$$

If an infected individual in a partnership dies, the partnership dissolves. Thus, an SI_i partnership dissolves at per-partnership rate λ_i, and the susceptible partner enters the single susceptible compartment at rate $\lambda_i SI_i$ (due to the SIS formulation, the infected partner that dies also gives rise, at an equal rate, to an individual entering the single susceptible compartment):

$$
\begin{aligned}
\text{DM}(S) &= \sum_k 2\lambda_k SI_k, \\
\text{DM}(SI_i) &= -\lambda_i SI_i.
\end{aligned} \tag{10}
$$

Similarly, since II_{ij} partnerships are composed of two infected partners, they dissolve at a per-partnership rate $(\lambda_i + \lambda_j)$. However, two cases, when $i \neq j$ and $i = j$, must be considered separately. When an II_{ij} partnership dissolves due to disease-induced mortality, where $i \neq j$, the death of the partner with strain i causes its partner to enter I_j compartment

at rate $\lambda_j II_{ij}$, and vice versa. When an $II_i i$ partnership dissolves, the death of either partner causes the other partner to enter the I_i compartment at rate $\lambda_i II_{ii}$, which sums up to $2\lambda_i II_{ii}$. Combining these dynamics yields:

$$\begin{aligned}
\mathrm{DM}(S) &= \sum_l \sum_k 2^{\delta_{lk}} \lambda_k II_{lk}, \\
\mathrm{DM}(I_i) &= \sum_k 2^{\delta_{ik}} \lambda_k II_{ik}, \\
\mathrm{DM}(II_{ij}) &= -(\lambda_i + \lambda_j) II_{ij}.
\end{aligned} \tag{11}$$

Finally, combining all these equations give us the full model, which is Model 1. We can simply drop the uncoupled and extra-couple transmission terms to obtain model 2:

$$\begin{aligned}
S' =& -\rho S + 2cSS + \sum_k cSI_k - \sum_k P_k U_k S + \sum_k \lambda_k I_k \\
& + \sum_k 2\lambda_k SI_k + \sum_l \sum_k 2^{\delta_{lk}} \lambda_k II_{lk} \\
I_i' =& -\rho I_i + cSI_i + \sum_k 2^{\delta_{ik}} cII_{ik} + \sum_k M_{ki} P_k U_k S - \lambda_i I_i \\
& + \sum_k 2^{\delta_{ik}} \lambda_k II_{ik} \\
SS' =& \frac{\rho S \cdot S}{2(S + \sum_k I_k)} - cSS - \sum_i 2P_i E_i SS \\
SI_i' =& \frac{\rho S \cdot I_i}{S + \sum_k I_k} - cSI_i - \beta_i SI_i + \sum_k 2M_{ki} P_k E_k SS - \sum_k P_k E_k SI_i \\
& - \lambda_i SI_i \\
II_{ij}' =& \left(\frac{1}{2}\right)^{\delta_{ij}} \cdot \frac{\rho I_i \cdot I_j}{(S + \sum_k I_k)} - cII_{ij} + \left(\frac{1}{2}\right)^{\delta_{ij}} \cdot (M_{ij}\beta_i SI_i + M_{ji}\beta_j SI_j) \\
& + \left(\frac{1}{2}\right)^{\delta_{ij}} \cdot (\sum_k (M_{kj} P_k E_k SI_i + M_{ki} P_k E_k SI_j)) - (\lambda_i + \lambda_j) II_{ij}
\end{aligned} \tag{12}$$

Models 3 ("instswitch") and 4 ("instswitch")

Partnership dynamics

Since model 3 and 4 assume instantaneous partnership formation, there are only three states: SS, SI_i, and II_{ij}. Partnership dissolution rates are equal to those of model 1 and 2: $\mathrm{DS}(SS) = -cSS$, $\mathrm{DS}(SI_i) = -cSI_i$, and $\mathrm{DS}(II_{ij}) = -cII_{ij}$. Once individuals leave a partnership, they are instantaneously distributed into coupled states. In order to make

the equations simpler, we introduce the following two terms: X and Y_i, where X denotes the total rate at which susceptible individuals leave partnership at a given time, and Y_i is the total partnership leaving rate of infected individuals with $\log_{10}$ SPVL of α_i at a given time. These temporarily single individuals then form couples through the same partnership formation rules described in the previous section:

$$\begin{aligned} X &= 2cSS + \sum_k cSI_k \\ Y_i &= cSI_i + \sum_k 2^{\delta_{ik}} cII_{ik} \\ SS' &= -cSS + \frac{X^2}{2(X + \sum_k Y_k)} \\ SI_i' &= -cSI_i + \frac{XY_i}{X + \sum_k Y_k} \\ II_{ij}' &= -cII_{ij} + \left(\frac{1}{2}\right)^{\delta_{ij}} \frac{Y_i Y_j}{X + \sum_k Y_k}. \end{aligned} \tag{13}$$

Instantaneous-switching models: Infection dynamics

Model 3 and 4 share the within-couple transmission term with model 1 and 2. Since there is no single (uncoupled) state, only extra-couple transmission occurs:

$$P_i = \frac{r_e(SI_i + \sum_k (II_{ik} + \delta_{ik} II_{ik}))}{r_e(2SS + \sum_k 2SI_k + \sum_l \sum_k (2^{\delta}_{kl} II_{lk}))}. \tag{14}$$

Movement from SS state to SI state and SI to SS is modeled through the same equations that are used in models 1 and 2.

Instantaneous-switching models: Disease induced mortality

Disease-induced mortality is modeled similarly to model 1 and 2. However, as the single state does not exist in model 3 and 4, individuals that leave partnerships due to death of their partners form partnerships instantly:

$$
\begin{aligned}
X &= \sum_k 2\lambda_k SI_k + \sum_l \sum_k 2^{\delta_{lk}} \lambda_k II_{lk}, \\
Y_i &= \sum_k 2^{\delta_{ik}} \lambda_k II_{ik}, \\
SS' &= = \frac{X^2}{2(X + \sum_k Y_k)}, \\
SI_i' &= -\lambda_i SI_i + \frac{XY_i}{X + \sum_k Y_k}, \\
II_{ij}' &= -(\lambda_i + \lambda_j) II_{ij} + \left(\frac{1}{2}\right)^{\delta_{ij}} \cdot \frac{Y_i Y_j}{X + \sum_k Y_k}.
\end{aligned} \tag{15}
$$

Combining all these dynamics, we have model 3. If we remove extra-couple transmission, we have model 4.

$$
\begin{aligned}
X =& 2cSS + \sum_k cSI_k + \sum_k 2\lambda_k SI_k + \sum_l \sum_k 2^{\delta_{lk}} \lambda_k II_{lk}, \\
Y_i =& cSI_i + \sum_k 2^{\delta_{ik}} cII_{ik} + \sum_k 2^{\delta_{ik}} \lambda_k II_{ik}, \\
SS' =& - cSS + \frac{X^2}{2(X + \sum_k Y_k)} - \sum_i 2P_i E_i SS, \\
SI_i' =& - cSI_i + \frac{XY_i}{X + \sum_k Y_k} - \beta_i SI_i + \sum_k 2M_{ki} P_k E_k SS \\
& - \sum_k P_k E_k SI_i - \lambda_i SI_i, \\
II_{ij}' & - cII_{ij} + \left(\frac{1}{2}\right)^{\delta_{ij}} \frac{Y_i Y_j}{X + \sum_k Y_k} + \left(\frac{1}{2}\right)^{\delta_{ij}} \cdot (M_{ij} \beta_i SI_i + M_{ji} \beta_j SI_j) \\
& + \left(\frac{1}{2}\right)^{\delta_{ij}} \cdot (\sum_k (M_{kj} P_k E_k SI_i + M_{ki} P_k E_k SI_j)) - (\lambda_i + \lambda_j) II_{ij}.
\end{aligned} \tag{16}
$$

Implicit model

Following Shirreff *et al.* [2], Model 5 is an implicit instantaneous partnership formation model that uses an adjusted transmission rate, β^*, that is derived from Hollingsworth *et al.*'s approximate basic reproduction number [3]:

$$
\beta_i^* = \frac{c\beta_i}{c + \beta_i + \lambda_i}. \tag{17}
$$

Thus, we get the following model:

$$
\begin{aligned}
S' &= \sum_k \lambda_k I_k - \sum_k \beta^*_k S I_k. \\
I'_i &= \sum_k M_{ki} \beta^*_k S I_k - \lambda_i I_i.
\end{aligned} \tag{18}
$$

Random-mixing model

Model 6 is a random-mixing model. It is modeled in a same way as model 5 without the adjusted transmission rate:

$$
\begin{aligned}
S' &= \sum_k \lambda_k I_k - \sum_k c\beta_k S I_k, \\
I'_i &= \sum_k M_{ki} c\beta_k S I_k - \lambda_i I_i.
\end{aligned} \tag{19}
$$

Heterogenous model

We extend the explicit models by allowing for heterogeneity in sexual behaviour. We first extend the "pairform+epc" model and obtain rest of the models by adjusting the heterogeneous "pairform+epc" model. Since pairform+epc model captures four distinct sexual behaviours – pair formation, pair dissolution, extra-couple mixing, and uncoupled mixing – we assume that all four parameters that model these behaviours are scaled by the same factor based on the risk group. In other words, an individual in a higher risk is also more likely to form a long-term sexual partnership, leave a long-term partnership, and engage in a extra-couple/uncoupled mixing. We denote this scaling parameter as φ_i where i is the risk group. For simplicity, we assume that the transmission rate per partnership is unaffected by sexual behaviour.

We allow for hetereogeneity in other three explicit models as well by adjusting the parameters of the heterogeneous "pairform+epc model". Heterogeneous "pairform model" is easily obtained by removing the extra-pair and uncoupled contact structure (setting $c_e/c_w = c_u/c_w = 0$, equivalently) from the heterogeneous "pairform+epc" model. We approximate heterogeneous "instswitch" and "instswitch+epc" models by setting the baseline pair formation rate, ρ_{base}, to a high value to let individuals to enter partnership almost instantaneously as soon as they achieve a single state. For "instswitch" model, both c_e/c_w and c_u/c_w were set to 0, whereas only c_u/c_w was set to 0 for "instswitch+epc" model. The following sections describe the heterogeneous "pairform+epc" model:

Partnership dynamics

Individuals in a risk group i leave single state at per-person rate $\varphi_i \rho$. Let $XY_{ij,kl}$ be a coupled state where X and Y are the infection status (susceptible or infected) of each partner, k and l are the risk groups X and Y belong to respectively, and i and j are the strains of an infected partner. If a partner is susceptible, strain index is replaced by $\cdot$. For example, $SI_{\cdot j,kl}$ is the number of partners where the susceptible partner is in risk group k and infected partner is in risk group l and has $\log_{10}$ SPVL of α_j. For simplicity, we assume that people undergo random *activity-weighted* mixing [4]. Then we can write the partnership formation process as follows:

$$\mathrm{PF}(XY_{ij,kl}) = \left(\frac{1}{2}\right)^{\delta_{ij}\delta_{kl}} \frac{\varphi_k \rho X_{i,k} \varphi_l \rho Y_{j,l}}{\sum\limits_m \varphi_m \rho (S_{\cdot,m} + \sum\limits_n I_{n,m})} \tag{20}$$

For dissolution process, an individual in risk group i leaves its partnership at a rate $\varphi_i c$. If a partnership is formed between two individuals from a different risk group, the rates at which they leave the partnership differ. We resolve this conflict by assuming that a partnership dissolution rate of a couple is equal to the average of that of two partners. Therefore, $XY_{ij,kl}$ dissolve at per-partnership rate $(\varphi_k + \varphi_l)/2c$, and both $X_{i,k}$ and $Y_{j,l}$ partners return to single state at the same rate [5].

Heterogeneous models: Infection dynamics

Since we assume that the rate of transmission per partnership stays constant across different risk groups, the within-couple infection process is similar to other models:

$$\begin{aligned} WT(SI_{\cdot j,kl} &= -\beta_j SI_{\cdot j,kl} \\ WT(II_{ij,kl}) &= \left(\frac{1}{2}\right)^{\delta_{ij}\delta_{kl}} \cdot (M_{ji}\beta_j SI_{\cdot j,kl} + M_{ij}\beta_i SI_{\cdot i,lk}) \end{aligned} \tag{21}$$

Note that $II_{ij,kl}$ can be formed from two types of partnerships: 1) Infected partner with $\log_{10}$ SPVL of α_j and risk group of l infects a susceptible partner in risk group k, yielding $\log_{10}$ SPVL of α_i through mutation. 2) Infected partner with $\log_{10}$ SPVL of α_i and risk group of k infected a susceptible partner in risk group l, yielding $\log_{10}$ SPVL of α_j through mutation. On the other hand, if $i = j$ and $k = l$, $II_{ii,kk}$ can only be formed from $SI_{\cdot i,kk}$ partnership, which is resolved by $\left(\frac{1}{2}\right)^{\delta_{ij}\delta_{kl}}$.

The heterogeneous extra-couple and uncoupled contact process is similar to the partnership formation process (activity-weighted random mixing). Relative uncoupled/extra-couple contact rates are scaled by the factor of φ_i, where i is the risk group. First, we define Q_i as the total rate of uncoupled/extra couple contact by individuals in risk group k:

$$\begin{aligned} Q_i =& \varphi_i r_u (S_{\cdot,i} + \sum_j I_{j,i}) + \varphi_i r_e \Bigg(\sum_k 2^{\delta_{ik}} SS_{\cdot,ik} + \\ & \sum_l \sum_j (SI_{\cdot j,il} + SI_{\cdot j,li}) + \sum_j \sum_l \sum_k 2^{\delta_{kl}\delta_{ij}} II_{kl,ij} \Bigg) \end{aligned} \tag{22}$$

We now define $P_{k,i}$ as the proportion of the extra-couple and uncoupled contact that arises from an infected individual from risk group i with $\log_{10}$ SPVL of α_k:

$$P_{k,i} = \frac{\varphi_i r_u I_{k,i} + \varphi_i r_e (SI_{k,i} + \sum_j \sum_l 2^{\delta_{kl}\delta_{ij}} II_{kl,ij})}{\sum_j Q_j} \tag{23}$$

Since the relative uncoupled/extra couple contact ratios are scaled by the factor of φ_i, uncoupled and extra-couple transmission rates are scaled by the same factor as well: $U_{k,i} = \varphi_i r_u \beta_k$ and $E_{k,i} = \varphi_i r_e \beta_k$. Once again, we assume activity-weighted mixing between individuals. Then, a susceptible individual in risk group i becomes infected through extra-couple and uncoupled contact at a per capita rate of $\sum_j \sum_k P_{k,j} X_{k,i}$. Once infected, individuals are distributed into strain categories through mutation.

Heterogeneous model: Disease induced mortality

Disease induced mortality is not affected by the sexual behaviour of an individual.

Vital dynamics

We extend four explicit models by adding vital dynamics and removing the SIS assumption). We follow Champredon *et al.*'s [1] and assume that all individuals suffer from per-capita death rate of m and that new susceptibles are born at a constant rate. Since we remove SIS assumption, individuals that die from disease induced mortality are simply removed from the population. Therefore, changes in the population due to birth and (both natural and disease induced) death in "pairform+epc" model can be written as follows (note that infection and partnership formation/dissolution processes have been ommitted in the equations for clarity):

$$
\begin{aligned}
S' &= m(1-S) + \sum_k \lambda_k SI_k + 2mSS + \sum_k mSI_k \\
I_i' &= -\lambda_i I_i + \sum_k 2^{\lambda_{ik}} \lambda_k II_{ik} - mI_i + mSI_k + \sum_k 2^{\lambda_{ik}} mII_{ik} \\
SS' &= -2mSS \\
SI_i' &= -\lambda_i SI_i - 2mSI_i \\
II_{ij}' &= -(\lambda_i + \lambda_j) II_{ij} - 2mII_{ij}
\end{aligned}
$$

By adding partnership dynamics and infection process, we obtain a "pairform+epc" model with vital dynamics. Other three explicit models with vital dynamics is obtained by adjusting the paraemters as discussed in the previous section.

Initial distribution of infected individuals

We follow Champredon *et al.*'s result to calculate the initial distribution of infected individuals [1]. For model 1 and 2, we have disease equilibrium state of $S^* = \frac{c}{c+\rho}$ and $SS^* = \frac{1-S^*}{2}$. We let $\epsilon = 10^{-4}$, which is the total number of infected individuals in the beginning of simulation and D be the vector such that D_i represent the proportion of individuals with $\log_{10}$ SPVL of i. D_i is taken from the Gaussian distribution with mean 3 and standard deviation 0.2 and is normalized so that $\sum_i D_i = 1$. Then, we have the following initial distribution of each states:

$$
\begin{aligned}
S(0) &= (1-\epsilon)S^*, \\
SS(0) &= (1-\epsilon)^2 SS^*, \\
SI_i(0) &= 2\epsilon(1-\epsilon)SS^* D_i, \\
I_i(0) &= \epsilon S^* D_i, \\
II_{ij}(0) &= \left(\frac{1}{2}\right)^{\delta_{ij}} 2\epsilon^2 SS^* D_i D_j.
\end{aligned} \tag{24}
$$

Note that we use this same approach for models with vital dynamics to calculate the initial distribution of each states after redefining $S^* = \frac{c+2m}{c+\rho+2m}$.

Since model 3 and 4 do not have single states, $SS^* = 1$ at the disease free equilibrium and the initial distribution becomes:

$$\begin{aligned} SS(0) &= (1-\epsilon)^2 SS^*, \\ SI_i(0) &= 2\epsilon(1-\epsilon) SS^* D_i, \\ II_{ij}(0) &= \left(\frac{1}{2}\right)^{\delta_{ij}} 2\epsilon^2 SS^* D_i D_j. \end{aligned} \tag{25}$$

As model 5 is an implicit model, which does not consider different stages of partnership, we have the following initial distribution:

$$\begin{aligned} S(0) &= 1-\epsilon, \\ I_i(0) &= \epsilon D_i. \end{aligned} \tag{26}$$

Model 6 has the same distribution of initial infected individuals as model 5.

Lastly, for the heterogeneity model, we assume that the risk distribution of the population follows a Gamma distribution and calculate the shape and scale parameters from the mean and squared coefficient of variation. Using the shape and scale parameters, we define the Gamma quantile function $Q(p)$ and $p_j = p_{\min} + (p_{\max} - p_{\min})\frac{j-1}{n_r+1}$, where n_r is number of risk groups and $j = 1, 2, 3, \ldots, n_r + 1$. Since $Q(1) = \infty$, we set $p_{\max} = 0.99$ and $p_{\min} = 0.01$. Then, we define $\varphi_i = \frac{Q(p_j)+Q(p_{j+1})}{2}$. We define R_i as the proportion of individuals in risk group i at the disease-free equilibrium and assume R_i is equal for all i, i.e. $R_i = \frac{1}{n_r}$. In order to start the simulation in a quasi-equilibrium state, we first run the model with the following initial state:

$$\begin{aligned} S_{\cdot,i}(0) &= (1-\epsilon) R_i, \\ I_{k,i}(0) &= \epsilon D_k R_i, \\ SS_{\cdot,ij}(0) &= SI_{\cdot k,ij}(0) = II_{kl,ij}(0) = 0. \end{aligned} \tag{27}$$

For this particular simulation, we disregard the infection process as well as disease-induced mortality in order to preserve the strain distribution of infected individuals. Furthermore, since the scaling parameter, γ, does not affect the risk group distribution in the absence of disease transmission, we increase the scaling parameter to 5 ($\gamma = 5$) to speed up the simulation and run the model for 50 years. After the model has reached its quasi-equilibrium state, we take this distribution of susceptible and infected individuals as the initial state of the actual simulation.

References

1. Champredon D, Bellan S, Dushoff J. HIV Sexual Transmission Is Predominantly Driven by Single Individuals Rather than Discordant Couples: A Model-Based Approach. PLoS ONE. 2013;8(12):e82906. doi:10.1371/journal.pone.0082906.

2. Shirreff G, Pellis L, Laeyendecker O, Fraser C. Transmission Selects for HIV-1 Strains of Intermediate Virulence: A Modelling Approach. PLoS Computational Biology. 2011;7(10):e1002185. doi:10.1371/journal.pcbi.1002185.

3. Hollingsworth TD, Anderson RM, Fraser C. HIV-1 Transmission, by Stage of Infection. Journal of Infectious Diseases. 2008;198(5):687–693. doi:10.1086/590501.

4. May RM, Anderson RM. The Transmission Dynamics of Human Immunodeficiency Virus (HIV) [and Discussion]. Philosophical Transactions of the Royal Society of London Series B, Biological Sciences. 1988;321(1207):565–607.

5. Herbeck JT, Mittler JE, Gottlieb GS, Goodreau SM, Murphy JT, Cori A, et al. Evolution of HIV virulence in response to widespread scale up of antiretroviral therapy: a modeling study. Virus Evolution. 2016;2(2):vew028. doi:10.1093/ve/vew028.
