# Supplementary material for: Effects of contact structure on the transient evolution of HIV virulence
Source: PLoS Comput Biol. 2017 Mar 31;13(3):e1005453. doi: 10.1371/journal.pcbi.1005453 (PMC5391972; doi:10.1371/journal.pcbi.1005453)
Supplement: S2 Appendix — (PDF) [file pcbi.1005453.s002.pdf]

Supporting Information S2: dynamics of transmission and virulence

This section presents alternate versions of the figures from the main text showing time dynamics and summary statistics in terms of expected progression time to AIDS and per-year transmission probability rather than $\log_{10}$ SPVL.

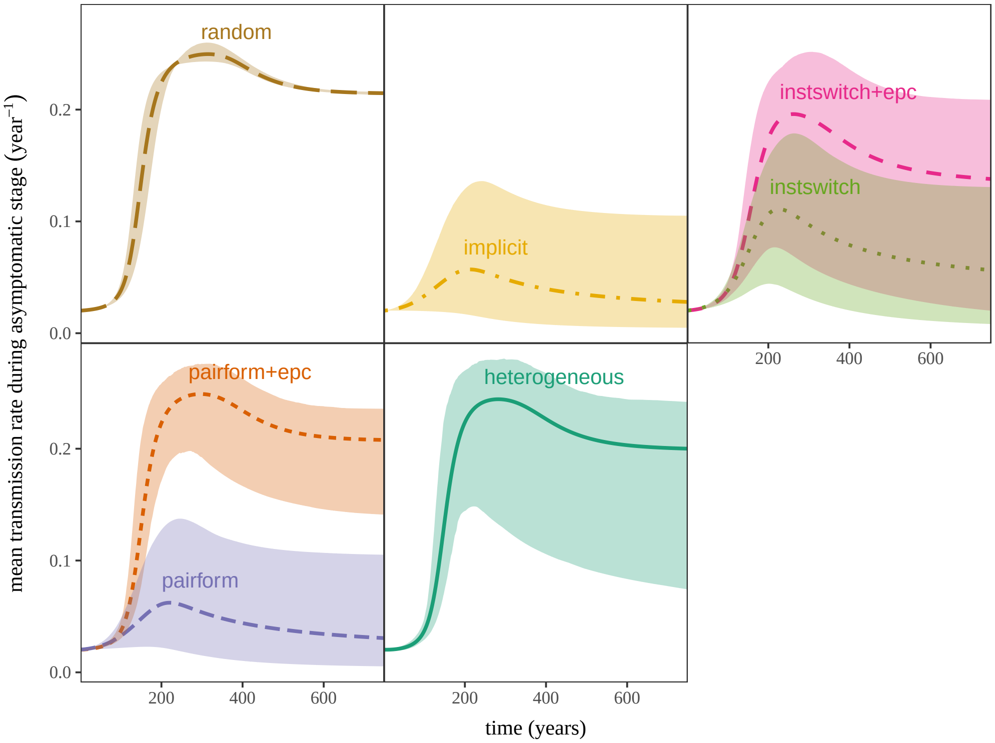

Fig A. Envelopes of transmission trajectories under all models. This figure matches Fig 3 in main text, but displays the envelope of population-mean transmission probabilities rather than mean $\log_{10}$ SPVL over time for each model.

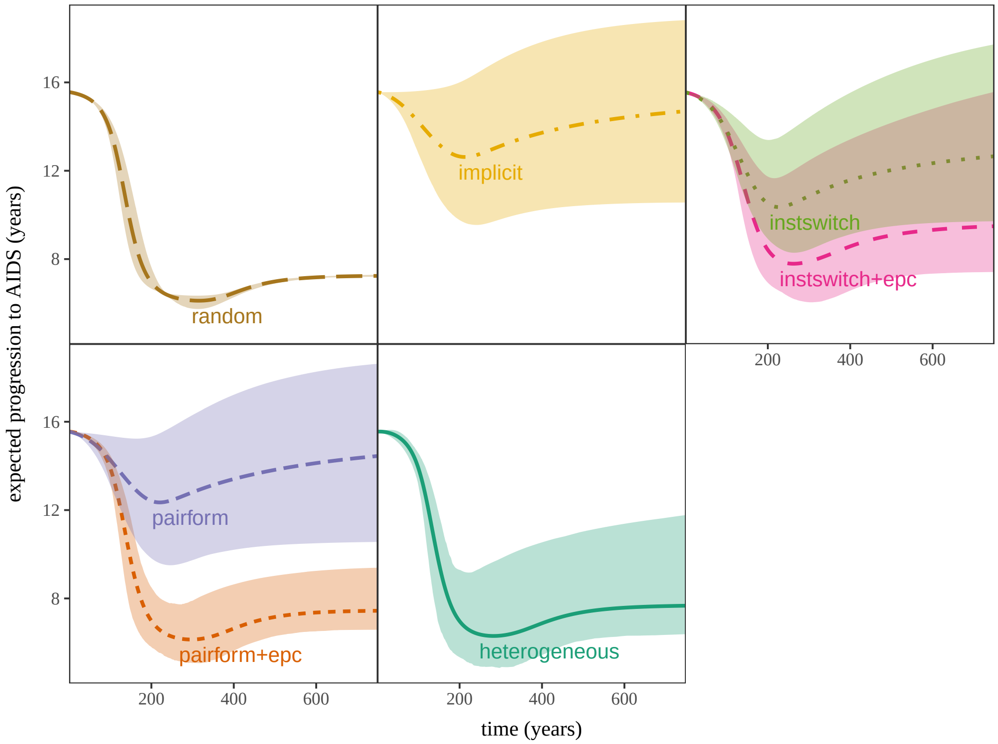

Fig B. Envelopes of expected progression time to AIDS trajectories under all models. This figure matches Fig 3 in main text, but displays the envelope of expected progression time to AIDS rather than mean $\log_{10}$ SPVL over time for each model.

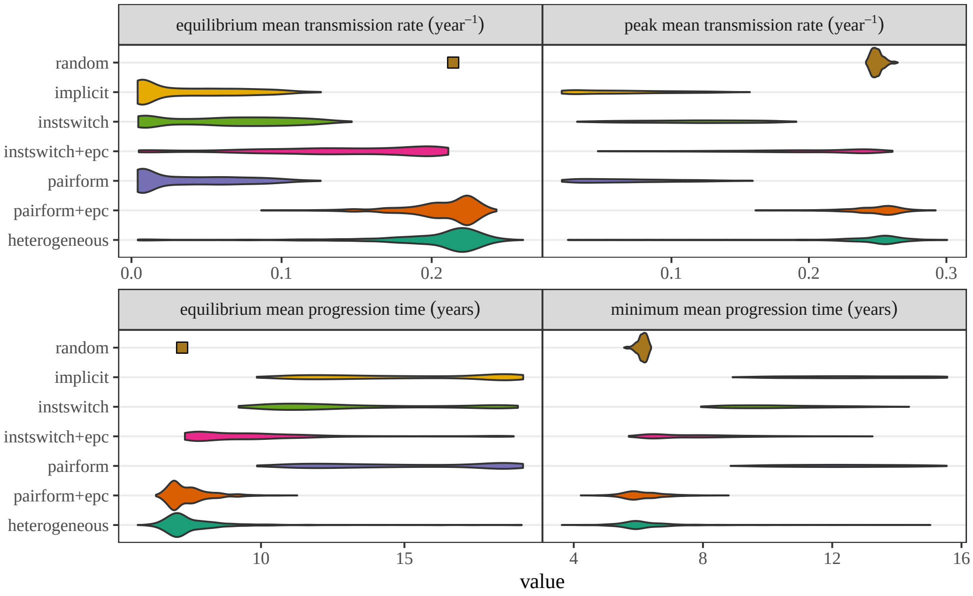

Fig C. Univariate distributions of transmission probabilities and expected progression time to AIDS. This figure matches Fig 4 in main text, but displays the univariate distributions for the transmission probability and expected progression time to AIDS time at the virulence peak and at the epidemiological equilibrium, rather than the distributions of $\log_{10}$ SPVL.

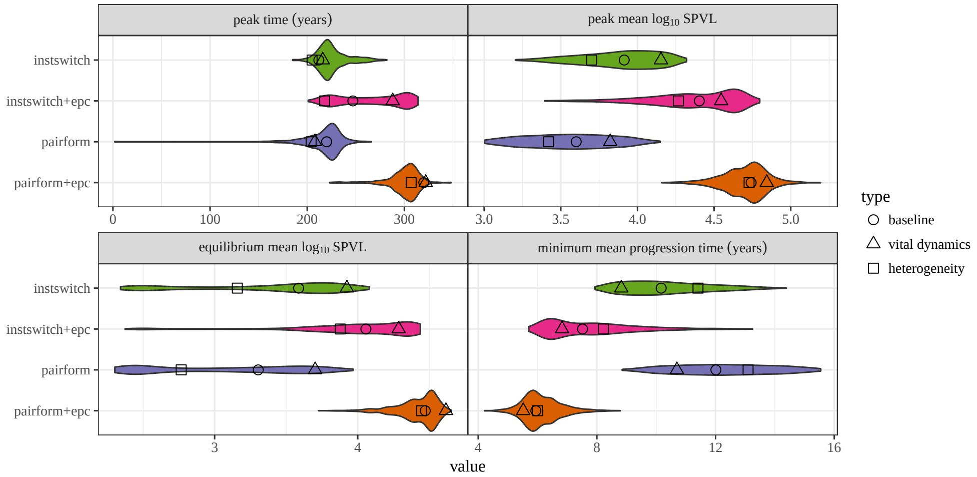

Fig D. Effects of contact heterogeneity and vital dynamics. This figure matches Fig 4 in main text, but adds results for model variants with contact heterogeneity and vital dynamics. The violin plots, copied from Fig 4 in the main text, show the distribution of outcomes across the Latin hypercube sample for SIS model without contact heterogeneity. The points show the outcome of various models using the baseline parameters from Table 1: (*circle*) unmodified models (SIS, homogeneous); (*triangle*) models with vital dynamics; (*square*) models with contact heterogeneity.
